# Association of the sarcopenia index with incident depressive symptoms and adverse depressive symptom trajectories

**DOI:** 10.3389/fnut.2026.1749859

**Published:** 2026-05-12

**Authors:** Xin Cai, Shaoqin Zhang, Zuqiao Zhao, Tianzuo Lan, Lili Mei, Qianqian Dai, Yingying Jin

**Affiliations:** 1 The First People’s Hospital of Guiyang, Guiyang, China; 2 Orthopedics Department, Taizhou Hospital of Zhejiang Province, Linhai, China; 3 Emergency Department, Taizhou Hospital of Zhejiang Province, Linhai, China

**Keywords:** cohort study, depressive symptoms, muscle health, older adults, sarcopenia index, trajectories

## Abstract

**Background:**

The sarcopenia index (SI) is an accessible marker associated with muscle health, with cross-sectional link to depression has been reported. However, longitudinal evidence between baseline SI and future depressive symptoms is limited, without investigations on heterogeneous symptom trajectories. We intended to figure out the correlation of baseline SI and both the incidence of depressive symptoms and membership in distinct depressive symptom trajectories.

**Methods:**

Utilizing data from the Health and Retirement Study (2016–2022), our cohort included 6,286 participants free of depressive symptoms at baseline for the incident analysis and 5,699 for the trajectory analysis. Distinct symptom patterns were identified using group-based trajectory modeling (GBTM) across four waves. Associations were determined using Cox proportional hazards regression for incident depressive symptoms and multinomial logistic regression for trajectory membership.

**Results:**

Over the 6-year follow-up, 1,311 participants developed depressive symptoms (incidence rate: 47.73 per 1,000 person-years). Each standard deviation (SD) increase in baseline SI was connected to decreased likelihood of incident depressive symptoms (Hazard Ratio [HR] = 0.91; 95% Confidence Interval [CI]: 0.85–0.98; *p* = 0.008). When analyzed by quartiles, the highest quartile (Q4) was linked to diminished likelihood of incident depressive symptoms compared to the lowest quartile (Q1; HR = 0.80; 95% CI: 0.66–0.96; *p* = 0.018). Moreover, this association demonstrated a linear dose–response relationship among all subgroups and remained robust across sensitivity analyses. Furthermore, four distinct trajectories were identified by GBTM, including “Non-depressed” (28.09%), “Low-stable” (40.61%), “Moderate-progressive” (26.04%), and “High-progressive” (5.26%). A higher baseline SI was associated with significantly lower odds of belonging to the “High-progressive” trajectory (Per SD increase: Odds Ratio [OR] = 0.88; 95% CI: 0.80–0.97; *p* = 0.008). But the association was specific to adverse patterns, showing no association with the “Low-stable” trajectory (*p* = 0.973).

**Conclusion:**

Consistent association was observed between a higher baseline sarcopenia index and both a lower likelihood of incident depressive symptoms and a lower risk of membership in adverse symptom trajectories in older adults. These findings highlighted the utility of SI on identifying individuals at elevated risk for unfavorable long-term depressive symptom progression among older population.

## 1 Introduction

Depression is a debilitating condition that profoundly constrains psychosocial functioning, degrades overall quality of life, and imposes a substantial humanistic and economic burden on global health systems (1, 2). Currently, depression ranked as a leading cause of disease burden, which might become the primary cause worldwide in 2030 suggested by projections (1). This public health challenge is particularly pronounced among older adults (3), among which depressive symptoms are highly prevalent (4, 5). In addition, the clinical course of depressive disorders is often poorer and more chronic than in younger populations (6). A key feature of late-life depression is its complex interplay with declining physical health, which contribute to faster progression of multimorbidity complexity (7) and a higher prevalence of cardiometabolic diseases (8). Studies have consistently linked depressive symptoms to a higher likelihood of incident cardiovascular events (9, 10), significant functional impairments, and an elevated risk of falls in older adults (11). This association also extends to cognitive function, where depression may accentuate cognitive weaknesses (12, 13), and contributes to increased all-cause mortality (14). This intricate relationship between depressive symptoms and somatic decline, coupled with frequent underdiagnosis and undertreatment (15–17), highlights the critical need to identify objective physical index that may be associated with the development of depressive symptoms in older adults.

Among these objective physical indexes, sarcopenia has emerged as a key factor of interest. Sarcopenia is a clinical condition defined by the progressive decline in skeletal muscle mass, strength, and functional capacity (18). A substantial body of evidence from meta-analyses and cohort studies indicates an association between sarcopenia and depression. Specifically, individuals with sarcopenia not only have a high prevalence of depressive symptoms (18), but also face a higher likelihood of developing incident depressive symptoms (19–21). Moreover, certain markers have been identified, with low muscle strength independently correlated with future depression (22, 23). However, quantifying sarcopenia in large-scale epidemiological research is impeded by methodological barriers (24). Traditional diagnostic methods rely on device-dependent measurements, such as dual-energy X-ray absorptiometry or bioelectrical impedance analysis. These methods are often costly, time-consuming, and involve radiation exposure, so they are impractical for widespread screening in clinical practice or research field (25). These limitations have necessitated the need for accessible, objective, and cost-effective serum biomarkers (24, 26). The sarcopenia index (SI) has emerged as a viable surrogate marker (27, 28), which is frequently defined as the serum creatinine-to-cystatin C (Cr/CysC) ratio. This index is grounded in the biological premise that serum creatinine is predominantly influenced by muscle mass, whereas cystatin C remains largely independent of it (25). The SI has demonstrated moderate-to-high diagnostic accuracy for sarcopenia (26, 29), which aligns with gold-standard measurements of muscle mass (24, 30, 31) and related functional outcomes (25, 32).

Given its utility as a validated surrogate marker, recent research has increasingly explored the connection of SI and health outcomes. Longitudinal studies have associated a lower SI with a higher likelihood of adverse physical outcomes, including incident cardiovascular disease (24), all-cause mortality (33, 34), frailty (35), and cognitive impairment (36). However, whether SI is correlated with key mental health outcomes, particularly depressive symptoms, remains less clear. While some cross-sectional analyses have investigated SI and depressive symptoms, their findings have been inconsistent with results varying by sex (37) or involving complex mediation pathways (38). Crucially, the literature exploring the longitudinal association between SI and future incident depressive symptoms is limited, with outcome defined the statically (39). This approach fails to capture the heterogeneous nature of depression over time especially in older adults, which may follow distinct patterns such as worsening, improving, or persistently high trajectories (40–42). Characterizing these different trajectories is critical, as they are associated with different risks for subsequent severe health outcomes, including dementia and mortality (39, 40). To date, it remains unknown whether baseline SI, as an objective marker of muscle health, is associated with these distinct longitudinal patterns of depressive symptoms.

This study first examined the longitudinal correlation of baseline SI and the likelihood of incident depressive symptoms over a 6-year follow-up. We further identified distinct longitudinal patterns of depressive symptoms to determine the association between baseline SI and membership in these specific trajectory groups.

## 2 Methods

### 2.1 Data source and participant selection

The analysis collected data from the Health and Retirement Study (HRS), an ongoing, nationally representative longitudinal study of US adults aged 50 years and older (43). Conducted by the University of Michigan, the HRS has conducted biennial follow-up interviews since 1992. Our study cohort was derived from the 2016 wave, utilizing biomarker measurements of 2016 Venous Blood Study (VBS). All participants who finished the 2016 core interview were invited to participate in the VBS, with the exception of those residing in nursing homes and individuals requiring a proxy respondent. A total of 20,912 respondents were identified in the 2016 HRS wave.

**Figure 1:**
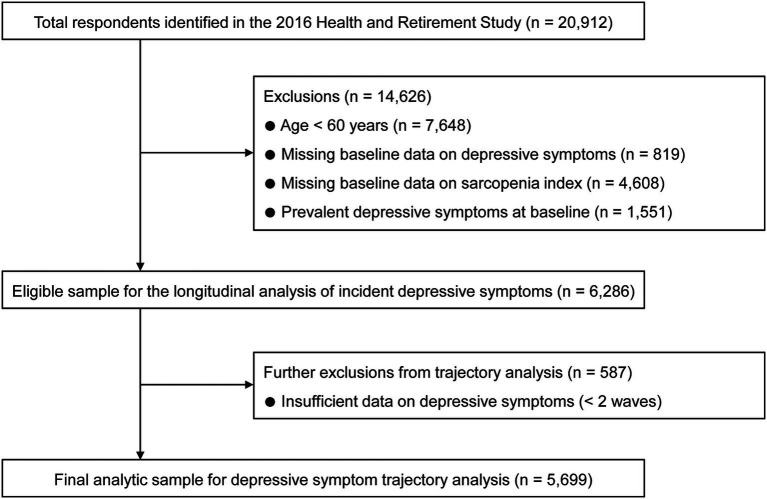
Flowchart of the study participant selection.

The participant selection process is detailed in Figure 1. For the analysis of incident depressive symptoms, we established several exclusion criteria. We excluded 7,648 individuals younger than 60 years, 819 participants with missing baseline data on depressive symptoms, and 4,608 participants missing the sarcopenia index. To ensure the cohort was at risk for new-onset depressive symptoms, 1,551 participants with prevalent depressive symptoms at baseline were also excluded, yielding an eligible sample of 6,286 participants for the longitudinal analysis. For the depressive symptom trajectory analysis, we further excluded 587 individuals who had insufficient data on depressive symptoms (defined as < 2 waves), resulting in a final analytic cohort of 5,699 participants. The HRS protocol was reviewed and approved by the Institutional Review Board at the University of Michigan (Reference No. HUM00061128), and written informed consent was obtained from all participants.

### 2.2 Sarcopenia index measurement

Quantification of the SI was based on serum analytes collected in 2016 as part of the VBS, a component of the HRS. These samples were collected during an at-home visit by a trained phlebotomist and shipped overnight to the CLIA-certified Advanced Research and Diagnostic Laboratory at the University of Minnesota. Laboratory records indicate 99% of specimens were processed within 48 h of collection. Serum creatinine was quantified on a Roche COBAS 6000 analyzer using a Roche enzymatic method. This assay is traceable to the National Institute of Standards and Technology reference material SRM 909b and demonstrated an inter-assay coefficient of variation (CV) of 2.9% at 0.835 mg/dL. On the same platform, serum cystatin C was measured using a Gentian particle-enhanced turbidimetric immunoassay, which had an inter-assay CV of 4.3% at 0.75 mg/L. The SI was calculated as (serum creatinine / cystatin C) × 100 (24).

### 2.3 Definition of depressive symptom outcomes

Depressive symptomatology was evaluated by the 8-item Center for Epidemiologic Studies Depression Scale (CES-D) (44). This instrument provides a total score (range 0–8) based on dichotomous (yes/no) responses to eight items. Two positive items (“was happy,” “enjoyed life”) are reverse-coded before summation, with elevated scores denoting more severe depressive symptomatology. The CES-D scores were used to define two distinct outcomes. For the longitudinal analysis, incident depressive symptoms were defined by a CES-D score ≥3 at any follow-up wave (2018, 2020, or 2022), restricted to participants free of depressive symptoms (CES-D < 3) at the 2016 baseline. For the trajectory analysis, the CES-D scores from all four waves (2016–2022) were used to model patterns of symptom change over time.

### 2.4 Measurement of potential confounders

Several potential confounders were ascertained at the 2016 baseline, primarily from the HRS core interview. Age, body mass index (BMI), and serum vitamin D (ng/mL) from the VBS were treated as continuous variables, with BMI calculated from self-reported height and weight. Sociodemographic information was categorized as follows: sex (male or female), ethnicity (Non-Hispanic or Hispanic), marital status (married or other), and education level (less than upper secondary, upper secondary and vocational, or tertiary). Self-reported lifestyle factors were dichotomized (no or yes), including smoking status, alcohol consumption, and moderate physical activity. Moderate physical activity was categorized as yes if participants reported engaging in moderately energetic activities at least once per week and as no otherwise. Clinical information was based on self-reported physician diagnoses (i.e., “Has a doctor ever told you that you had...”). This included the history of hypertension, dyslipidemia, diabetes, arthritis, stroke, heart disease, and cancer (all categorized as no or yes).

### 2.5 Statistical analysis

Baseline features of participants were summarized in line with quartiles of the SI. Continuous variables were presented as mean ± standard deviation (SD) and categorical variables as number (percentage). Differences across groups were assessed using one-way analysis of variance for continuous variables and chi-square test for categorical variables.

To assess the association of baseline SI and incident depressive symptoms, we employed Cox regression, with findings reported as hazard ratios (HR) and 95% confidence intervals (CI). Verification of the proportional hazard assumption was performed using Schoenfeld residual testing. Prior to adjustment, multicollinearity among covariates was assessed utilizing variance inflation factor (VIF), with all values indicating no serious collinearity (VIF < 5). For these models, SI was included both as a continuous variable and as quartiles (the lowest quartile [Q1] as reference). A P for trend was determined by modeling the median value as a continuous term. We constructed three sequential models: Model 1 was unadjusted; Model 2 was adjusted for age, sex, ethnicity, and education level; and Model 3 was further adjusted for marital status, smoking status, alcohol consumption, BMI, moderate physical activity, hypertension, dyslipidemia, diabetes, arthritis, stroke, heart disease, and vitamin D. Restricted cubic splines (RCS) with four knots were used to examine the potential for a non-linear dose–response relationship.

Distinct patterns of depressive symptoms over time were identified using group-based trajectory modeling (GBTM) (45). This analysis was restricted to participants with at least two CES-D measurements (*n* = 5,699), utilizing scores from the 2016, 2018, 2020, and 2022 waves. We fitted models with one to six trajectories using a censored normal distribution and selected the optimal number of groups based on several criteria: the Akaike and the Bayesian Information Criterion, an average posterior probability (AvePP) > 0.70 for each group, and each class containing at least 5% of the sample. Following identification of the trajectories, multinomial logistic regression was completed to examine the connection of baseline SI and the possibility of membership in each trajectory group, using the “Non-depressed” group as reference. These models were adjusted using the same three-model strategy as the Cox analyses.

Several additional analyses were conducted to assess robustness. Subgroup analyses were performed for both the incident depressive symptoms and the trajectory analysis, and P for interaction values were also calculated to evaluate potential effect modification. Sensitivity analyses for the incident depressive symptoms outcome included: (1) excluding individuals who developed depressive symptoms within the first 2 years of follow-up; (2) complete-case analysis; (3) a competing risk analysis using the Fine-Gray subdistribution hazard model, treating all-cause mortality as the competing event; (4) excluding participants with baseline cancer; (5) using a discrete-time survival model with a complementary log–log link; (6) using sex-specific quartiles of the sarcopenia index; and (7) further adjusting for estimated glomerular filtration rate based on creatinine and cystatin C (eGFRcr-cys). For the trajectory analysis, additional sensitivity analyses were performed by (1) excluding participants with baseline cancer and (2) further adjusting for eGFRcr-cys.

All statistical analyses were conducted in R software (version 4.4.1). For all tests, statistical significance was determined as *p* < 0.05 (two-sided).

## 3 Results

### 3.1 Baseline characteristics

Baseline characteristics for the 6,286 participants in the incident depressive symptoms cohort are presented in Supplementary Table S8, stratified by quartiles (Q1–Q4) of the sarcopenia index. The mean age of this cohort was 71.98 ± 8.44 years, and 3,483 (55.41%) were female. Significant differences were found across the quartiles for nearly all baseline variables, with the exception of dyslipidemia (*p* = 0.581). Compared to participants in the highest quartile (Q4, >93.28), those in the lowest quartile (Q1, <69.34) were generally older (mean age: 75.00 ± 9.00 vs. 68.90 ± 7.07 years) and included more female individuals (84.22% vs. 22.72%). Furthermore, patients in Q1 had a higher mean body mass index (29.46 ± 6.82 vs. 27.86 ± 4.80 kg/m2), a lower prevalence of moderate physical activity (55.39% vs. 79.89%), and a higher prevalence of comorbidities, including hypertension (71.56% vs. 59.45%), diabetes (31.62% vs. 24.68%), and arthritis (73.85% vs. 53.24%).

Stratification of the 5,699 participants in the trajectory analysis by the four identified depressive symptom groups also revealed significant differences for most baseline characteristics (Supplementary Table S8). For example, compared with “Non-depressed” group, participants in “High-progressive” group were more possibly to be female (67.00% vs. 49.84%) and Hispanic (20.00% vs. 9.19%). Additionally, individuals in “High-progressive” group also presented a lower proportion of individuals with tertiary education (47.67% vs. 62.77%) and a higher prevalence of several comorbidities at baseline, including arthritis (77.33% vs. 53.47%) and heart disease (35.00% vs. 20.86%).

### 3.2 Association between SI and depressive symptoms

**Figure 2:**
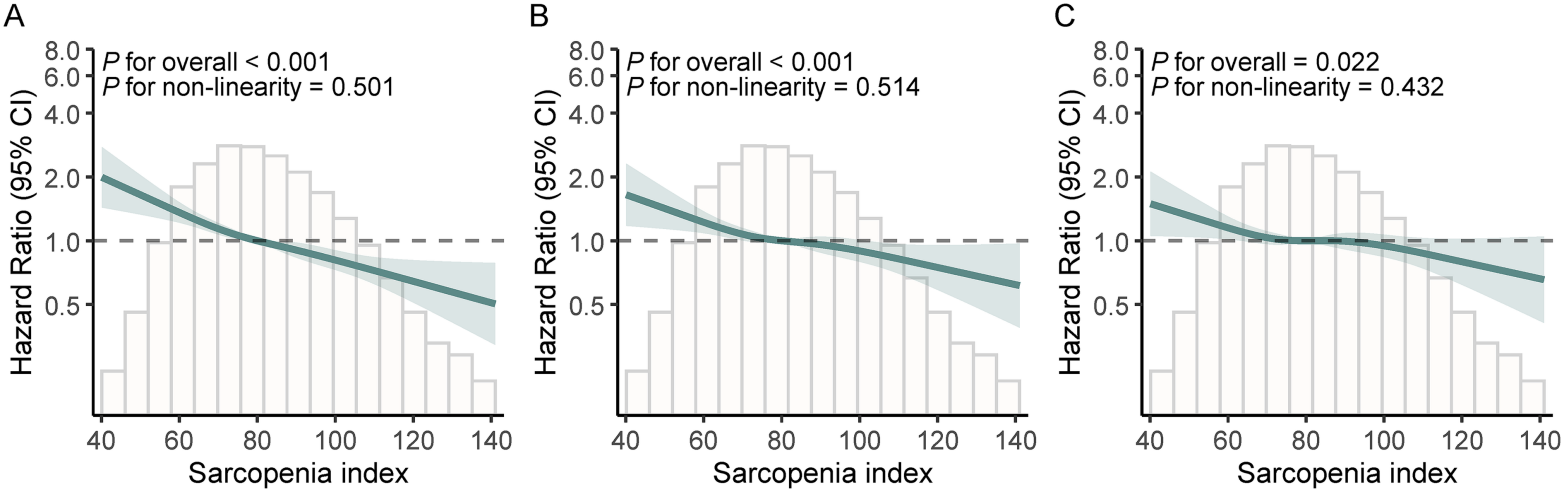
Dose–response relationship between sarcopenia index and the risk of incident depressive symptoms. Panels **A–C** correspond to Model 1, Model 2, and Model 3, respectively. Model 1 was unadjusted. Model 2 was adjusted for age, sex, ethnicity, and education level. Model 3 was further adjusted for marital status, smoking status, alcohol consumption, body mass index, moderate physical activity, hypertension, dyslipidemia, diabetes, arthritis, stroke, heart disease, and vitamin D.

**Figure 3:**
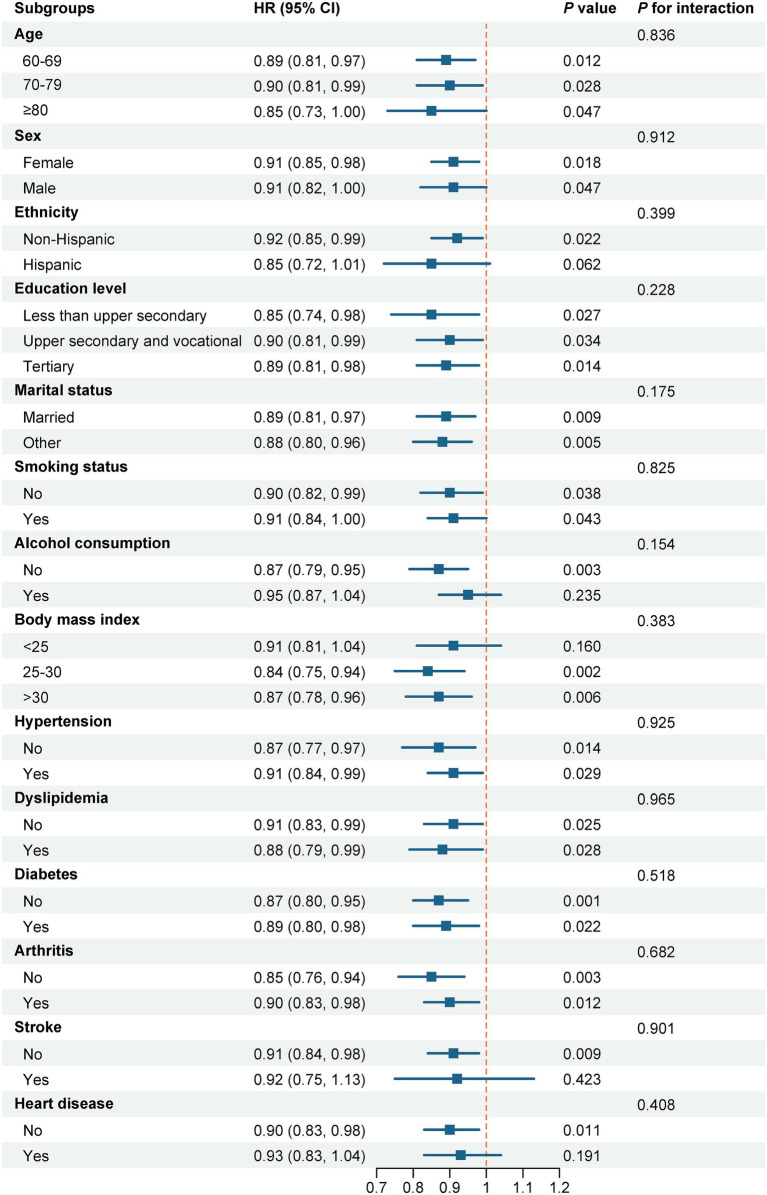
Subgroup analysis of the association between sarcopenia index and the risk of incident depressive symptoms. Note: The figure shows the hazard ratios (HRs) and 95% confidence intervals (CIs) for the association between the sarcopenia index (per 1-SD increase) and incident depressive symptoms stratified by potential risk factors. Each subgroup analysis was adjusted for the same covariates as in the fully adjusted model (Model 3), except for the stratification variable itself.

Over the follow-up period, 1,311 participants developed depressive symptoms, corresponding to an incidence rate of 47.73 per 1,000 person-years (Table 1). In Model 3, each SD increase in baseline SI was connected to a diminished likelihood of incident depressive symptoms (HR = 0.91; 95% CI: 0.85–0.98; *p* = 0.008). When SI was analyzed by quartiles, participants in Q4 had a decreased likelihood of incident depressive symptoms in contrast to individuals in Q1 (HR = 0.80; 95% CI: 0.66–0.96; *p* = 0.018), with a significant trend revealed among quartiles (P for trend = 0.031). The RCS analysis additionally suggested a linear dose–response relationship, with no evidence of non-linearity (Figure 2; P for non-linearity = 0.432 in Model 3). Also, this association was consistent across all subgroups examined in Figure 3 (all P for interaction > 0.154).

**Table 1:** Association of baseline sarcopenia index with the risk of incident depressive symptoms.

Exposure	Events, n	Total, n	Incidence rate	Model 1		Model 2		Model 3	
				HR (95% CI)	*p* value	HR (95% CI)	*p* value	HR (95% CI)	*p* value
Per SD increase	1,311	6,286	47.73	0.81 (0.76, 0.86)	<0.001	0.87 (0.82, 0.93)	<0.001	0.91 (0.85, 0.98)	0.008
Quartiles									
Q1	386	1,572	64.42	Ref		Ref		Ref	
Q2	330	1,571	48.26	0.76 (0.65, 0.88)	<0.001	0.82 (0.70, 0.95)	0.009	0.88 (0.75, 1.04)	0.125
Q3	323	1,567	45.58	0.72 (0.62, 0.83)	<0.001	0.82 (0.70, 0.96)	0.011	0.85 (0.73, 0.99)	0.042
Q4	272	1,576	36.03	0.57 (0.48, 0.66)	<0.001	0.70 (0.59, 0.85)	<0.001	0.80 (0.66, 0.96)	0.018
*P* for trend					<0.001		<0.001		0.031

Incidence rates are presented per 1,000 person-years. Model 1 was unadjusted. Model 2 was adjusted for age, sex, ethnicity, and education level. Model 3 was further adjusted for marital status, smoking status, alcohol consumption, body mass index, moderate physical activity, hypertension, dyslipidemia, diabetes, arthritis, stroke, heart disease, and vitamin D.

Furthermore, the results were robust in sensitivity analyses. Similar associations were observed after excluding cases occurring within the first 2 years of follow-up (Supplementary Table S1; HR = 0.90; 95% CI: 0.82–0.99; *p* = 0.037), in the complete-case analysis (Supplementary Table S2; HR = 0.90; 95% CI: 0.84–0.96; *p* = 0.003), and in the competing-risk analysis with all-cause mortality treated as a competing event (Supplementary Table S3; SHR = 0.92; 95% CI: 0.87–0.99; *p* = 0.016). Further adjustment for eGFRcr-cys yielded similar results (per SD increase: HR = 0.90; 95% CI: 0.84–0.96; p = 0.003; Q4 vs. Q1: HR = 0.77; 95% CI: 0.64–0.93; *p* = 0.006; P for trend = 0.011). Similar results were also observed after excluding participants with baseline cancer (Supplementary Table S4), using a discrete-time survival model (Supplementary Table S5), and applying sex-specific quartiles of the sarcopenia index (Supplementary Table S6).

### 3.3 Associations between SI and trajectories of depressive symptoms

**Figure 4:**
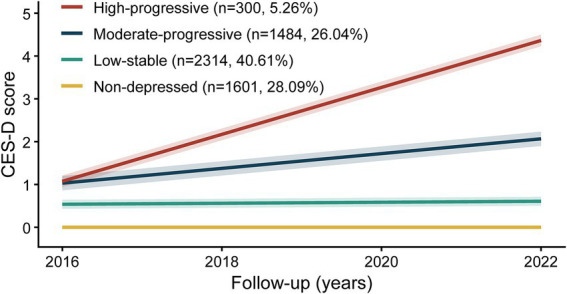
Identified trajectories of depressive symptoms from 2016 to 2022.

Four distinct depressive symptom trajectories were identified through GBTM analysis among 5,699 participants, a model supported by fit statistics (Supplementary Table S7). As shown in Figure 4, these groups were “Non-depressed” (28.09%), “Low-stable” (40.61%), “Moderate-progressive” (26.04%), and “High-progressive” (5.26%).

The associations between baseline SI and trajectory membership were presented in Table 2, using the “Non-depressed” group as the reference category. In Model 3, a greater baseline SI was linked to lower possibility of belonging to “High-progressive” group, which was observed when SI was considered as a continuous variable (Each SD increase: OR = 0.88; 95% CI: 0.80–0.97; *p* = 0.008) and by quartiles (Q4 vs. Q1: OR = 0.71; 95% CI: 0.55–0.92; *p* = 0.010), with a significant trend (P for trend = 0.017). A similar pattern was noted for the “Moderate-progressive” trajectory, where participants in Q4 had lower possibility of membership compared to Q1 (OR = 0.62; 95% CI: 0.39–0.97; *p* = 0.037; P for trend = 0.026). While there is a trend for statistical significance when considering SI as a continuous variable (OR = 0.85; 95% CI: 0.71–1.00; *p* = 0.057). Nevertheless, no correlation has been found of baseline SI and membership in the “Low-stable” trajectory (Each SD increase: OR = 1.00; 95% CI: 0.92–1.08; *p* = 0.973). In addition, these associations were consistent across all tested subgroups (Supplementary Table S9; all P for interaction > 0.05). Similar results were observed after excluding participants with baseline cancer (Supplementary Table S10) and after further adjustment for eGFRcr-cys (Supplementary Table S11).

**Table 2:** Association of sarcopenia index with depressive symptom trajectories.

Model	Low-stable		Moderate-progressive		High-progressive	
	OR (95% CI)	*p* value	OR (95% CI)	*p* value	OR (95% CI)	*p* value
Model 1						
Per SD increase	0.90 (0.84–0.95)	<0.001	0.67 (0.59–0.77)	<0.001	0.72 (0.67–0.78)	<0.001
Quartiles						
Q1	Ref		Ref		Ref	
Q2	0.88 (0.73–1.07)	0.202	0.57 (0.40–0.80)	0.001	0.72 (0.59–0.88)	0.002
Q3	0.82 (0.68–0.99)	0.043	0.46 (0.33–0.66)	<0.001	0.62 (0.50–0.75)	<0.001
Q4	0.68 (0.57–0.82)	<0.001	0.33 (0.23–0.47)	<0.001	0.41 (0.33–0.50)	<0.001
*P* for trend		<0.001		<0.001		<0.001
Model 2						
Per SD increase	0.96 (0.89–1.03)	0.241	0.80 (0.68–0.94)	0.008	0.80 (0.73–0.87)	<0.001
Quartiles						
Q1	Ref		Ref		Ref	
Q2	0.95 (0.78–1.15)	0.568	0.67 (0.47–0.95)	0.025	0.81 (0.66–1.00)	0.049
Q3	0.92 (0.76–1.13)	0.425	0.61 (0.42–0.88)	0.009	0.75 (0.60–0.93)	0.008
Q4	0.81 (0.65–1.00)	0.051	0.51 (0.33–0.79)	0.002	0.55 (0.43–0.70)	<0.001
*P* for trend		0.054		0.001		<0.001
Model 3						
Per SD increase	1.00 (0.92–1.08)	0.973	0.85 (0.71–1.00)	0.057	0.88 (0.80–0.97)	0.008
Quartiles						
Q1	Ref		Ref		Ref	
Q2	1.01 (0.83–1.23)	0.939	0.73 (0.51–1.05)	0.090	0.91 (0.73–1.14)	0.417
Q3	1.03 (0.83–1.26)	0.805	0.70 (0.47–1.03)	0.073	0.92 (0.73–1.16)	0.473
Q4	0.92 (0.73–1.15)	0.456	0.62 (0.39–0.97)	0.037	0.71 (0.55–0.92)	0.010
*P* for trend		0.484		0.026		0.017

The “Non-depressed” trajectory group served as the reference outcome category. Model 1 was unadjusted. Model 2 was adjusted for age, sex, ethnicity, and education level. Model 3 was further adjusted for marital status, smoking status, alcohol consumption, body mass index, moderate physical activity, hypertension, dyslipidemia, diabetes, arthritis, stroke, heart disease, and vitamin D.

## 4 Discussion

This longitudinal cohort study examined associations between the sarcopenia index and two distinct depressive symptom outcomes in older adults. While SI was utilized as a serum-based surrogate for muscle health, its baseline level was correlated with a diminished likelihood of incident depressive symptoms. Furthermore, the analysis of longitudinal symptom patterns found that a higher SI was also linked to a decreased likelihood of belonging to trajectories characterized by worsening or persistently high depressive symptoms. However, this association appeared specific to these adverse trajectories, as no linkage was observed for stable, low-level symptom patterns. Moreover, these associations persisted after adjustment for a comprehensive range of potential confounders and remained consistent across several sensitivity analyses.

The finding of an inverse association between baseline SI and incident depressive symptoms provides a crucial longitudinal extension to previous reports, which were predominantly cross-sectional and yielded inconsistent results. For example, one prior cross-sectional study utilizing dataset from CHARLS observed an association between SI and depressive symptoms only in males (37). In contrast, our study observed this longitudinal association in the overall HRS cohort, without evidence of effect modification by sex according to subgroup analyses. These differing observations might be related to differences in study design, including cross-sectional or longitudinal assessment, as well as variations in population characteristics. Despite this, our main finding is highly consistent with the broader body of evidence linking sarcopenia to depressive symptoms. Meta-analyses (46) and other prospective studies (20, 21, 47) using device-based definitions have reported similar associations, which has also found for components like low muscle strength (23) and genetically proxied lean mass (48). Previous longitudinal research on the SI has largely focused on its predicting role for physical health, with a lower SI linked to a higher likelihood of somatic outcomes including cardiovascular disease (24), frailty (35), and all-cause mortality (34). The present study therefore contributes new evidence suggesting its role not only for muscle health but also for long-term course of a key mental health outcome.

Beyond the binary definition of incidence, this study also firstly examined longitudinal patterns of depressive symptoms caused by SI. The use of group-based trajectory modeling is well-established in depression research and has been applied to identify heterogeneous symptom courses in diverse older populations, ensuring robustness of our findings (49–51). Previous work has successfully linked these trajectories to a wide array of correlates, including functional disability (52), metabolic indices (Triglyceride-Glucose index) (53), and psychosocial determinants such as spirituality (54) or childhood experiences (55). However, the relationship with serum-based surrogates of muscle health has yet to be explored. This approach is crucial, as much of the existing literature on depression risk has been based on a binary definition assessed at a single time point. But such static assessment (39, 56) may not fully capture the heterogeneity inherent in the course of late-life depression, which was addresses by identification of a nuanced pattern in our analyses. A lower SI was linked to higher possibility of membership in the adverse “Moderate-progressive” and “High-progressive” trajectories, which has not been confirmed in “Low-stable” group. This suggests that the physiological processes reflected by the SI may be associated not only with the initial onset of depressive symptoms but also, perhaps more specifically, with the distinct patterns of symptom progression over time.

The observational design of this study does not permit inferences regarding underlying mechanisms (57). However, previous literature suggested that low muscle mass and depression may share common biological antecedents, which was mediated by chronic low-grade inflammation. This inflammatory state was recognized as a contributor to muscle atrophy in sarcopenia (58, 59). Concurrently, the “cytokine hypothesis” of depression posited that these pro-inflammatory mediators are central to the pathophysiology of muscle metabolism and neuroendocrine dysregulation (60). Hyperactivity of the hypothalamic–pituitary–adrenal (HPA) axis was frequently observed in depression (61, 62) and results in elevated glucocorticoids, primarily contributing to muscle catabolism (63, 64). This broader state of metabolic disturbance also included insulin resistance, which was independently associated with both impaired muscle protein synthesis (65, 66) and adverse brain function (67). On the other hand, skeletal muscle also functioned as an endocrine organ (68), as reduced SI implied decreased muscle mass and a consequently altered secretome such as irisin, which was reported to influence neuroplasticity (69, 70). The association observed in our study may thus be indirect, reflecting the complex interplay of these shared biological systems.

This investigation has several strengths, including the longitudinal design within a large, nationally representative cohort, the use of an objective serum biomarker for muscle health, and the application of two complementary depressive symptom outcomes (incidence and trajectories). The consistency of the findings across multiple sensitivity analyses further reinforces our observations. However, some shortcomings must be addressed. The observational design precludes causal inferences and cannot eliminate the possibility of residual confounding. Reverse causality also cannot be fully excluded, as underlying metabolic disorders, baseline depressive symptoms below the CES-D cutoff, or age-related anorexia may have influenced the sarcopenia index. Detailed dietary factors, particularly total energy and protein intake, were not included in the present analysis and may have confounded the observed associations. There are also some measurement limitations. Depressive symptoms were evaluated using the CES-D screening scale rather than a structured clinical interview. While this may have introduced outcome misclassification, the CES-D is a widely validated and frequently utilized instrument in large-scale epidemiological research (44). Additionally, exclusions due to missing data may have introduced selection bias. However, the associations remained consistent in a complete-case sensitivity analysis, suggesting no substantial alteration of the findings. Furthermore, the SI and covariates were measured only at baseline, precluding an examination of whether changes in SI were associated with depressive symptom trajectories over time. Finally, the findings are derived from a cohort of older US adults. Therefore, whether these observations could be applied to younger individuals or populations in other settings remains to be determined. These findings suggest that SI may have potential value as an accessible and low-cost serum-based marker for identifying older adults at elevated risk of persistent or worsening depressive symptoms. Future longitudinal studies should validate these findings in diverse populations, incorporate repeated measurements of SI, and further examine whether interventions targeting muscle health may influence long-term depressive symptom trajectories.

## 5 Conclusion

In this large, longitudinal cohort of older US adults, a higher baseline sarcopenia index was associated with a lower likelihood of incident depressive symptoms over a 6-year follow-up. This association extended to longitudinal symptom patterns, as a higher baseline SI was also associated with lower odds of membership in trajectories characterized by adverse symptom progression. SI may have potential value as an accessible serum-based marker of long-term depressive symptom risk in older adults.

## Data Availability

Publicly available datasets were analyzed in this study. This data can be found via the Health and Retirement Study website at https://hrs.isr.umich.edu.
