# Supplementary material for: Association of the sarcopenia index with incident depressive symptoms and adverse depressive symptom trajectories
Source: Front Nutr. 2026 May 12;13:1749859. doi: 10.3389/fnut.2026.1749859 (PMC13201189; doi:10.3389/fnut.2026.1749859)
Supplement: Supplementary file 1 [file Table_1.docx]

**Table S1.** Sensitivity analysis of sarcopenia index and incident depressive symptoms excluding cases within the first 2 years

| **Exposure** | **Model 1** |  | **Model 2** |  | **Model 3** |  |
| --- | --- | --- | --- | --- | --- | --- |
|  | **HR (95% CI)** | ***P* value** | **HR (95% CI)** | ***P* value** | **HR (95% CI)** | ***P* value** |
| Per SD increase | 0.80 (0.74, 0.87) | <0.001 | 0.87 (0.79, 0.96) | 0.004 | 0.90 (0.82, 0.99) | 0.037 |
| Quartiles |  |  |  |  |  |  |
| Q1 | Ref |  | Ref |  |  |  |
| Q2 | 0.78 (0.64, 0.96) | 0.016 | 0.89 (0.72, 1.10) | 0.273 | 0.94 (0.76, 1.18) | 0.614 |
| Q3 | 0.78 (0.64, 0.95) | 0.014 | 0.85 (0.69, 1.05) | 0.129 | 0.87 (0.70, 1.07) | 0.192 |
| Q4 | 0.56 (0.45, 0.69) | <0.001 | 0.70 (0.55, 0.90) | 0.006 | 0.78 (0.60, 1.01) | 0.056 |
| *P* for trend |  | <0.001 |  | <0.001 |  | 0.001 |

Note: Model 1 was unadjusted. Model 2 was adjusted for age, sex, ethnicity, and education level. Model 3 was further adjusted for marital status, smoking status, alcohol consumption, body mass index, moderate physical activity, hypertension, dyslipidemia, diabetes, arthritis, stroke, heart disease, and vitamin D.

**Table S2.** Sensitivity analysis of sarcopenia index and incident depressive symptoms excluding missing data

| **Exposure** | **Model 1** |  | **Model 2** |  | **Model 3** |  |
| --- | --- | --- | --- | --- | --- | --- |
|  | **HR (95% CI)** | ***P* value** | **HR (95% CI)** | ***P* value** | **HR (95% CI)** | ***P* value** |
| Per SD increase | 0.80 (0.76, 0.85) | <0.001 | 0.86 (0.80, 0.93) | <0.001 | 0.90 (0.84, 0.96) | 0.003 |
| Quartiles |  |  |  |  |  |  |
| Q1 | Ref |  | Ref |  | Ref |  |
| Q2 | 0.75 (0.65, 0.87) | <0.001 | 0.81 (0.70, 0.94) | 0.007 | 0.87 (0.74, 1.02) | 0.082 |
| Q3 | 0.71 (0.61, 0.82) | <0.001 | 0.80 (0.68, 0.94) | 0.006 | 0.86 (0.74, 1.00) | 0.052 |
| Q4 | 0.56 (0.48, 0.66) | <0.001 | 0.69 (0.58, 0.83) | <0.001 | 0.77 (0.64, 0.93) | 0.008 |
| *P* for trend |  | <0.001 |  | <0.001 |  | 0.002 |

Note: Model 1 was unadjusted. Model 2 was adjusted for age, sex, ethnicity, and education level. Model 3 was further adjusted for marital status, smoking status, alcohol consumption, body mass index, moderate physical activity, hypertension, dyslipidemia, diabetes, arthritis, stroke, heart disease, and vitamin D.

**Table S3.** Sensitivity analysis of sarcopenia index and incident depressive symptoms considering competing risk of death

| **Exposure** | **Model 1** |  | **Model 2** |  | **Model 3** |  |
| --- | --- | --- | --- | --- | --- | --- |
|  | **SHR (95% CI)** | ***P* value** | **SHR (95% CI)** | ***P* value** | **SHR (95% CI)** | ***P* value** |
| Per SD increase | 0.86 (0.82, 0.91) | <0.001 | 0.91 (0.85, 0.97) | 0.003 | 0.92 (0.87, 0.99) | 0.016 |
| Quartiles |  |  |  |  |  |  |
| Q1 | Ref |  | Ref |  | Ref |  |
| Q2 | 0.83 (0.72, 0.96) | 0.010 | 0.88 (0.76, 1.02) | 0.085 | 0.90 (0.77, 1.05) | 0.200 |
| Q3 | 0.81 (0.70, 0.93) | 0.003 | 0.87 (0.76, 1.00) | 0.057 | 0.89 (0.77, 1.03) | 0.120 |
| Q4 | 0.67 (0.58, 0.78) | <0.001 | 0.78 (0.66, 0.93) | 0.005 | 0.82 (0.69, 0.98) | 0.025 |
| *P* for trend |  | <0.001 |  | 0.008 |  | 0.037 |

Note: SHR, subdistribution hazard ratio. Subdistribution hazard ratios and 95% confidence intervals were estimated using Fine-Gray subdistribution hazard models, with all-cause mortality treated as a competing event. Model 1 was unadjusted. Model 2 was adjusted for age, sex, ethnicity, and education level. Model 3 was further adjusted for marital status, smoking status, alcohol consumption, body mass index, moderate physical activity, hypertension, dyslipidemia, diabetes, arthritis, stroke, heart disease, and vitamin D.

**Table S4.** Sensitivity analysis of sarcopenia index and incident depressive symptoms after excluding baseline cancer

| **Exposure** | **Model 1** |  | **Model 2** |  | **Model 3** |  |
| --- | --- | --- | --- | --- | --- | --- |
|  | **HR (95% CI)** | ***P* value** | **HR (95% CI)** | ***P* value** | **HR (95% CI)** | ***P* value** |
| Per SD increase | 0.80 (0.75, 0.86) | <0.001 | 0.87 (0.80, 0.93) | <0.001 | 0.90 (0.84, 0.98) | 0.011 |
| Quartiles |  |  |  |  |  |  |
| Q1 | Ref |  | Ref |  | Ref |  |
| Q2 | 0.76 (0.65, 0.90) | 0.001 | 0.83 (0.70, 0.97) | 0.023 | 0.88 (0.74, 1.06) | 0.171 |
| Q3 | 0.72 (0.61, 0.85) | <0.001 | 0.82 (0.69, 0.97) | 0.022 | 0.87 (0.73, 1.03) | 0.096 |
| Q4 | 0.56 (0.48, 0.67) | <0.001 | 0.70 (0.57, 0.85) | 0.001 | 0.80 (0.65, 0.98) | 0.033 |
| *P* for trend |  | <0.001 |  | 0.001 |  | 0.049 |

Note: Model 1 was unadjusted. Model 2 was adjusted for age, sex, ethnicity, and education level. Model 3 was further adjusted for marital status, smoking status, alcohol consumption, body mass index, moderate physical activity, hypertension, dyslipidemia, diabetes, arthritis, stroke, heart disease, and vitamin D.

**Table S5.** Sensitivity analysis using a discrete-time survival model for the association of baseline sarcopenia index with incident depressive symptoms

| **Exposure** | **Model 1** |  | **Model 2** |  | **Model 3** |  |
| --- | --- | --- | --- | --- | --- | --- |
|  | **HR (95% CI)** | ***P* value** | **HR (95% CI)** | ***P* value** | **HR (95% CI)** | ***P* value** |
| Per SD increase | 0.80 (0.75, 0.85) | <0.001 | 0.87 (0.81, 0.93) | <0.001 | 0.90 (0.84, 0.97) | 0.005 |
| Quartiles |  |  |  |  |  |  |
| Q1 | Ref |  | Ref |  | Ref |  |
| Q2 | 0.74 (0.64, 0.86) | <0.001 | 0.81 (0.69, 0.94) | 0.005 | 0.87 (0.74, 1.02) | 0.085 |
| Q3 | 0.70 (0.60, 0.81) | <0.001 | 0.80 (0.68, 0.94) | 0.005 | 0.84 (0.72, 0.98) | 0.027 |
| Q4 | 0.55 (0.47, 0.64) | <0.001 | 0.69 (0.58, 0.83) | <0.001 | 0.79 (0.65, 0.95) | 0.013 |
| *P* for trend |  | <0.001 |  | <0.001 |  | 0.025 |

Note: HRs were estimated using a discrete-time survival model with a complementary log-log link. Model 1 was unadjusted. Model 2 was adjusted for age, sex, ethnicity, and education level. Model 3 was further adjusted for marital status, smoking status, alcohol consumption, body mass index, moderate physical activity, hypertension, dyslipidemia, diabetes, arthritis, stroke, heart disease, and vitamin D.

**Table S6.** Sensitivity analysis using sex-specific quartiles of the sarcopenia index for incident depressive symptoms

| **Exposure** | **Model 1** |  | **Model 2** |  | **Model 3** |  |
| --- | --- | --- | --- | --- | --- | --- |
|  | **HR (95% CI)** | ***P* value** | **HR (95% CI)** | ***P* value** | **HR (95% CI)** | ***P* value** |
| Quartiles |  |  |  |  |  |  |
| Q1 | Ref |  | Ref |  | Ref |  |
| Q2 | 0.92 (0.79, 1.07) | 0.280 | 0.96 (0.83, 1.12) | 0.644 | 0.97 (0.83, 1.12) | 0.649 |
| Q3 | 0.77 (0.66, 0.90) | 0.001 | 0.83 (0.71, 0.97) | 0.022 | 0.87 (0.74, 1.02) | 0.094 |
| Q4 | 0.68 (0.58, 0.80) | <0.001 | 0.75 (0.64, 0.89) | 0.001 | 0.82 (0.69, 0.98) | 0.026 |
| *P* for trend |  | <0.001 |  | <0.001 |  | 0.013 |

Note: Quartiles of the sarcopenia index were defined separately in men and women and then combined for analysis. Model 1 was unadjusted. Model 2 was adjusted for age, sex, ethnicity, and education level. Model 3 was further adjusted for marital status, smoking status, alcohol consumption, body mass index, moderate physical activity, hypertension, dyslipidemia, diabetes, arthritis, stroke, heart disease, and vitamin D.

**Table S7.** Model fit statistics for depressive symptom trajectories

| **Fit statistics** | **Number of classes** | |  |  |  |  |
| --- | --- | --- | --- | --- | --- | --- |
|  | **1** | **2** | **3** | **4** | **5** | **6** |
| AIC | 68417.62 | 52383.11 | -2225247.13 | -2225784.68 | -2225784.69 | -2225851.91 |
| BIC | 68441.37 | 52438.54 | -2225175.87 | -2225681.75 | -2225650.08 | -2225685.63 |
| AvePP | Class 1, 1.00 | Class 1, 0.93 | Class 1, 1.00 | Class 1, 1.00 | Class 1, 1.00 | Class 1, 1.00 |
|  |  | Class 2, 0.99 | Class 2, 0.95 | Class 2, 0.86 | Class 2, 0.94 | Class 2, 0.85 |
|  |  |  | Class 3, 0.95 | Class 3, 0.94 | Class 3, 0.86 | Class 3, 0.92 |
|  |  |  |  | Class 4, 0.89 | Class 4, 0.87 | Class 4, 0.84 |
|  |  |  |  |  | Class 5, 0.66 | Class 5, NA |
|  |  |  |  |  |  | Class 6, NA |
| Class proportion | Class 1, 100% | Class 1, 50.54% | Class 1, 28.09% | Class 1, 28.09% | Class 1, 28.09% | Class 1, 28.09% |
|  |  | Class 2, 49.46% | Class 2, 21.69% | Class 2, 26.04% | Class 2, 40.97% | Class 2, 24.13% |
|  |  |  | Class 3, 50.22% | Class 3, 40.61% | Class 3, 25.95% | Class 3, 40.94% |
|  |  |  |  | Class 4, 5.26% | Class 4, 4.93% | Class 4, 6.84% |
|  |  |  |  |  | Class 5, 0.06% | Class 5, NA |
|  |  |  |  |  |  | Class 6, NA |

Abbreviations: AIC, Akaike information criterion; AvePP, Average posterior probability; BIC, Bayesian information criterion.

**Table S8.** Baseline characteristics of study participants according to depressive symptom trajectories

| **Characteristics** | **Overall** | **Trajectory of depression** | |  |  | ***P* value** |
| --- | --- | --- | --- | --- | --- | --- |
|  |  | **Non-depressed** | **Low-stable** | **Moderate-progressive** | **High-progressive** |  |
| **Number, n** | 5699 | 1601 | 2314 | 1484 | 300 |  |
| **Age, years** | 71.47 ± 8.14 | 71.08 ± 8.00 | 71.26 ± 8.12 | 72.14 ± 8.21 | 71.88 ± 8.37 | <0.001 |
| **Body mass index, kg/m^2^** | 28.62 ± 5.67 | 28.16 ± 5.32 | 28.52 ± 5.49 | 29.20 ± 6.13 | 29.07 ± 6.15 | <0.001 |
| **Vitamin D, ng/mL** | 33.32 ± 13.23 | 33.92 ± 12.63 | 33.51 ± 13.57 | 32.71 ± 13.42 | 31.64 ± 12.52 | 0.008 |
| **eGFRcr-cys** | 72.84 ± 20.51 | 75.25 ± 19.94 | 73.24 ± 20.55 | 69.93 ± 20.61 | 71.08 ± 20.89 | <0.001 |
| **Sex, n (%)** |  |  |  |  |  | <0.001 |
| Male | 2507 (43.99) | 803 (50.16) | 1022 (44.17) | 583 (39.29) | 99 (33.00) |  |
| Female | 3192 (56.01) | 798 (49.84) | 1292 (55.83) | 901 (60.71) | 201 (67.00) |  |
| **Ethnicity, n (%)** |  |  |  |  |  | <0.001 |
| Non-Hispanic | 4997 (87.77) | 1452 (90.81) | 2024 (87.54) | 1281 (86.44) | 240 (80.00) |  |
| Hispanic | 696 (12.23) | 147 (9.19) | 288 (12.46) | 201 (13.56) | 60 (20.00) |  |
| **Education level, n (%)** |  |  |  |  |  | <0.001 |
| Less than upper secondary | 799 (14.02) | 119 (7.43) | 323 (13.97) | 301 (20.28) | 56 (18.67) |  |
| Upper secondary and vocational | 1876 (32.93) | 477 (29.79) | 790 (34.17) | 508 (34.23) | 101 (33.67) |  |
| Tertiary | 3022 (53.05) | 1005 (62.77) | 1199 (51.86) | 675 (45.49) | 143 (47.67) |  |
| **Marital status, n (%)** |  |  |  |  |  | <0.001 |
| Married | 3465 (60.85) | 1079 (67.48) | 1416 (61.22) | 813 (54.82) | 157 (52.51) |  |
| Other | 2229 (39.15) | 520 (32.52) | 897 (38.78) | 670 (45.18) | 142 (47.49) |  |
| **Smoking status, n (%)** |  |  |  |  |  | 0.016 |
| No | 2634 (46.43) | 775 (48.68) | 1086 (47.05) | 635 (43.08) | 138 (46.15) |  |
| Yes | 3039 (53.57) | 817 (51.32) | 1222 (52.95) | 839 (56.92) | 161 (53.85) |  |
| **Alcohol consumption, n (%)** |  |  |  |  |  | <0.001 |
| No | 2377 (41.73) | 579 (36.16) | 950 (41.05) | 708 (47.81) | 140 (46.67) |  |
| Yes | 3319 (58.27) | 1022 (63.84) | 1364 (58.95) | 773 (52.19) | 160 (53.33) |  |
| **Moderate physical activity, n (%)** |  |  |  |  |  | <0.001 |
| No | 1681 (29.58) | 328 (20.55) | 689 (29.84) | 569 (38.45) | 95 (31.88) |  |
| Yes | 4002 (70.42) | 1268 (79.45) | 1620 (70.16) | 911 (61.55) | 203 (68.12) |  |
| **Hypertension, n (%)** |  |  |  |  |  | <0.001 |
| No | 2075 (36.41) | 689 (43.04) | 869 (37.55) | 433 (29.18) | 84 (28.00) |  |
| Yes | 3624 (63.59) | 912 (56.96) | 1445 (62.45) | 1051 (70.82) | 216 (72.00) |  |
| **Dyslipidemia, n (%)** |  |  |  |  |  | <0.001 |
| No | 3726 (65.74) | 1125 (70.71) | 1518 (66.00) | 910 (61.61) | 173 (57.67) |  |
| Yes | 1942 (34.26) | 466 (29.29) | 782 (34.00) | 567 (38.39) | 127 (42.33) |  |
| **Diabetes, n (%)** |  |  |  |  |  | <0.001 |
| No | 4173 (73.22) | 1261 (78.76) | 1691 (73.08) | 1011 (68.13) | 210 (70.00) |  |
| Yes | 1526 (26.78) | 340 (21.24) | 623 (26.92) | 473 (31.87) | 90 (30.00) |  |
| **Arthritis, n (%)** |  |  |  |  |  | <0.001 |
| No | 2030 (35.62) | 745 (46.53) | 820 (35.44) | 397 (26.75) | 68 (22.67) |  |
| Yes | 3669 (64.38) | 856 (53.47) | 1494 (64.56) | 1087 (73.25) | 232 (77.33) |  |
| **Stroke, n (%)** |  |  |  |  |  | <0.001 |
| No | 5256 (92.23) | 1507 (94.13) | 2146 (92.74) | 1331 (89.69) | 272 (90.67) |  |
| Yes | 443 (7.77) | 94 (5.87) | 168 (7.26) | 153 (10.31) | 28 (9.33) |  |
| **Heart disease, n (%)** |  |  |  |  |  | <0.001 |
| No | 4218 (74.01) | 1267 (79.14) | 1728 (74.68) | 1028 (69.27) | 195 (65.00) |  |
| Yes | 1481 (25.99) | 334 (20.86) | 586 (25.32) | 456 (30.73) | 105 (35.00) |  |
| **Cancer, n (%)** |  |  |  |  |  | 0.147 |
| No | 4763 (83.58) | 1366 (85.32) | 1926 (83.23) | 1223 (82.41) | 248 (82.67) |  |
| Yes | 936 (16.42) | 235 (14.68) | 388 (16.77) | 261 (17.59) | 52 (17.33) |  |

Note: Data are presented as mean ± standard deviation for continuous variables and number (percentage) for categorical variables. P values were calculated using one-way analysis of variance for continuous variables and chi-square test for categorical variables.

**Table S9.** Subgroup analysis of the association between sarcopenia index and depressive symptom trajectories

| **Subgroups** | **Low-stable** |  | **Moderate-progressive** |  | **High-progressive** |  | ***P* for interaction** |
| --- | --- | --- | --- | --- | --- | --- | --- |
|  | **OR (95% CI)** | ***P* value** | **OR (95% CI)** | ***P* value** | **OR (95% CI)** | ***P* value** |  |
| **Age** |  |  |  |  |  |  | 0.773 |
| 60-69 | 0.97 (0.87-1.08) | 0.530 | 0.82 (0.65-1.04) | 0.106 | 0.87 (0.76-0.98) | 0.027 |  |
| 70-79 | 1.10 (0.95-1.27) | 0.199 | 0.89 (0.65-1.22) | 0.482 | 0.94 (0.80-1.11) | 0.486 |  |
| ≥80 | 0.99 (0.80-1.22) | 0.908 | 0.89 (0.59-1.33) | 0.559 | 0.82 (0.65-1.04) | 0.097 |  |
| **Sex** |  |  |  |  |  |  | 0.624 |
| Female | 1.06 (0.94-1.20) | 0.340 | 0.95 (0.75-1.20) | 0.672 | 0.94 (0.81-1.08) | 0.350 |  |
| Male | 0.96 (0.87-1.07) | 0.468 | 0.77 (0.60-1.00) | 0.047 | 0.84 (0.74-0.96) | 0.009 |  |
| **Ethnicity** |  |  |  |  |  |  | 0.727 |
| Non-Hispanic | 1.00 (0.92-1.09) | 0.919 | 0.83 (0.69-1.00) | 0.051 | 0.88 (0.80-0.97) | 0.012 |  |
| Hispanic | 0.96 (0.74-1.26) | 0.790 | 0.85 (0.53-1.37) | 0.510 | 0.87 (0.65-1.18) | 0.375 |  |
| **Education level** |  |  |  |  |  |  | 0.089 |
| Less than upper secondary | 1.21 (0.94-1.57) | 0.145 | 1.05 (0.69-1.62) | 0.810 | 0.97 (0.74-1.26) | 0.801 |  |
| Upper secondary and vocational | 0.94 (0.82-1.09) | 0.412 | 0.77 (0.57-1.04) | 0.091 | 0.91 (0.77-1.08) | 0.281 |  |
| Tertiary | 1.00 (0.90-1.11) | 0.978 | 0.80 (0.63-1.03) | 0.087 | 0.85 (0.75-0.97) | 0.016 |  |
| **Marital status** |  |  |  |  |  |  | 0.261 |
| Married | 1.03 (0.93-1.14) | 0.581 | 0.80 (0.63-1.01) | 0.058 | 0.89 (0.79-1.00) | 0.054 |  |
| Other | 0.97 (0.84-1.11) | 0.618 | 0.93 (0.72-1.20) | 0.586 | 0.87 (0.75-1.01) | 0.077 |  |
| **Smoking status** |  |  |  |  |  |  | 0.429 |
| No | 1.04 (0.92-1.17) | 0.529 | 0.73 (0.57-0.92) | 0.007 | 0.85 (0.75-0.96) | 0.011 |  |
| Yes | 0.97 (0.87-1.08) | 0.590 | 1.02 (0.79-1.32) | 0.860 | 0.91 (0.79-1.04) | 0.171 |  |
| **Alcohol consumption** |  |  |  |  |  |  | 0.633 |
| No | 0.99 (0.88-1.12) | 0.931 | 0.78 (0.61-0.99) | 0.043 | 0.85 (0.75-0.97) | 0.014 |  |
| Yes | 1.01 (0.91-1.12) | 0.888 | 0.91 (0.71-1.16) | 0.444 | 0.90 (0.79-1.04) | 0.152 |  |
| **Body mass index** |  |  |  |  |  |  | 0.219 |
| <25 | 1.05 (0.89-1.22) | 0.573 | 0.63 (0.43-0.92) | 0.017 | 0.79 (0.67-0.92) | 0.003 |  |
| 25-30 | 1.01 (0.89-1.14) | 0.912 | 1.01 (0.77-1.33) | 0.918 | 0.86 (0.74-1.01) | 0.061 |  |
| >30 | 0.96 (0.84-1.10) | 0.568 | 0.80 (0.60-1.05) | 0.113 | 1.03 (0.86-1.23) | 0.759 |  |
| **Hypertension** |  |  |  |  |  |  | 0.555 |
| No | 0.99 (0.87-1.12) | 0.886 | 0.80 (0.58-1.12) | 0.191 | 0.89 (0.79-1.00) | 0.049 |  |
| Yes | 1.01 (0.91-1.12) | 0.832 | 0.86 (0.70-1.06) | 0.156 | 0.87 (0.75-1.03) | 0.103 |  |
| **Dyslipidemia** |  |  |  |  |  |  | 0.491 |
| No | 1.00 (0.91-1.10) | 0.977 | 0.79 (0.64-0.99) | 0.043 | 0.88 (0.79-0.99) | 0.028 |  |
| Yes | 1.01 (0.87-1.16) | 0.942 | 0.92 (0.70-1.21) | 0.569 | 0.88 (0.75-1.04) | 0.135 |  |
| **Diabetes** |  |  |  |  |  |  | 0.211 |
| No | 1.01 (0.92-1.10) | 0.910 | 0.75 (0.61-0.93) | 0.008 | 0.86 (0.77-0.96) | 0.006 |  |
| Yes | 1.02 (0.87-1.21) | 0.780 | 1.10 (0.82-1.48) | 0.530 | 0.97 (0.81-1.16) | 0.705 |  |
| **Arthritis** |  |  |  |  |  |  | 0.257 |
| No | 0.93 (0.83-1.05) | 0.258 | 0.81 (0.58-1.13) | 0.209 | 0.87 (0.77-0.99) | 0.030 |  |
| Yes | 1.05 (0.94-1.17) | 0.362 | 0.87 (0.71-1.07) | 0.193 | 0.91 (0.78-1.06) | 0.228 |  |
| **Stroke** |  |  |  |  |  |  | 0.368 |
| No | 0.99 (0.91-1.08) | 0.850 | 0.82 (0.68-0.98) | 0.030 | 0.88 (0.80-0.97) | 0.008 |  |
| Yes | 1.20 (0.83-1.74) | 0.328 | 0.99 (0.55-1.77) | 0.978 | 0.93 (0.64-1.37) | 0.724 |  |
| **Heart disease** |  |  |  |  |  |  | 0.826 |
| No | 0.98 (0.90-1.08) | 0.739 | 0.83 (0.67-1.02) | 0.082 | 0.84 (0.75-0.94) | 0.002 |  |
| Yes | 1.07 (0.90-1.28) | 0.434 | 0.92 (0.67-1.24) | 0.571 | 1.00 (0.83-1.21) | 0.987 |  |

Note: The "Non-depressed" trajectory group served as the reference outcome category. Each subgroup analysis was adjusted for all covariates included in the fully adjusted model (Model 3), except for the stratification variable itself.

**Table S10**. Sensitivity analysis of the association between sarcopenia index and depressive symptom trajectories after excluding participants with baseline cancer

| **Model** | **Low-stable** |  | **Moderate-progressive** | | **High-progressive** |  |
| --- | --- | --- | --- | --- | --- | --- |
|  | **OR (95% CI)** | ***P* value** | **OR (95% CI)** | ***P* value** | **OR (95% CI)** | ***P* value** |
| **Model 1** |  |  |  |  |  |  |
| Per SD increase | 0.90 (0.84-0.96) | 0.002 | 0.69 (0.59-0.80) | <0.001 | 0.71 (0.66-0.77) | <0.001 |
| Quartiles |  |  |  |  |  |  |
| Q1 | Ref |  | Ref |  | Ref |  |
| Q2 | 0.98 (0.80-1.21) | 0.883 | 0.62 (0.43-0.91) | 0.015 | 0.77 (0.62-0.95) | 0.017 |
| Q3 | 0.87 (0.71-1.07) | 0.196 | 0.54 (0.37-0.79) | 0.001 | 0.61 (0.49-0.76) | <0.001 |
| Q4 | 0.70 (0.58-0.86) | <0.001 | 0.36 (0.25-0.54) | <0.001 | 0.40 (0.32-0.50) | <0.001 |
| *P* for trend |  | <0.001 |  | <0.001 |  | <0.001 |
| **Model 2** |  |  |  |  |  |  |
| Per SD increase | 0.97 (0.89-1.05) | 0.419 | 0.82 (0.69-0.98) | 0.033 | 0.79 (0.72-0.87) | <0.001 |
| Quartiles |  |  |  |  |  |  |
| Q1 | Ref |  | Ref |  | Ref |  |
| Q2 | 1.07 (0.87-1.32) | 0.536 | 0.74 (0.50-1.10) | 0.136 | 0.88 (0.70-1.10) | 0.248 |
| Q3 | 0.99 (0.80-1.23) | 0.934 | 0.71 (0.47-1.07) | 0.106 | 0.74 (0.59-0.94) | 0.015 |
| Q4 | 0.85 (0.68-1.08) | 0.183 | 0.59 (0.37-0.95) | 0.029 | 0.54 (0.42-0.70) | <0.001 |
| *P* for trend |  | 0.128 |  | 0.022 |  | <0.001 |
| **Model 3** |  |  |  |  |  |  |
| Per SD increase | 1.02 (0.93-1.11) | 0.708 | 0.87 (0.72-1.05) | 0.156 | 0.88 (0.79-0.97) | 0.014 |
| Quartiles |  |  |  |  |  |  |
| Q1 | Ref |  | Ref |  | Ref |  |
| Q2 | 1.15 (0.93-1.43) | 0.200 | 0.83 (0.54-1.28) | 0.396 | 0.99 (0.78-1.26) | 0.954 |
| Q3 | 1.11 (0.88-1.39) | 0.370 | 0.82 (0.55-1.22) | 0.327 | 0.91 (0.71-1.18) | 0.486 |
| Q4 | 0.97 (0.76-1.24) | 0.831 | 0.70 (0.53-0.93) | 0.013 | 0.71 (0.54-0.94) | 0.016 |
| *P* for trend |  | 0.677 |  | 0.016 |  | 0.013 |

Note: The "Non-depressed" trajectory group served as the reference outcome category. Model 1 was unadjusted. Model 2 was adjusted for age, sex, ethnicity, and education level. Model 3 was further adjusted for marital status, smoking status, alcohol consumption, body mass index, moderate physical activity, hypertension, dyslipidemia, diabetes, arthritis, stroke, heart disease, and vitamin D.

**Table S11**. Sensitivity analysis of the association between sarcopenia index and depressive symptom trajectories after additional adjustment for eGFR

| **Exposure** | **Low-stable** |  | **Moderate-progressive** | | **High-progressive** |  |
| --- | --- | --- | --- | --- | --- | --- |
|  | **OR (95% CI)** | ***P* value** | **OR (95% CI)** | ***P* value** | **OR (95% CI)** | ***P* value** |
| Per SD increase | 1.00 (0.93-1.08) | 0.959 | 0.85 (0.72-1.01) | 0.060 | 0.88 (0.80-0.97) | 0.009 |
| Quartiles |  |  |  |  |  |  |
| Q1 | Ref |  | Ref |  | Ref |  |
| Q2 | 1.01 (0.83-1.23) | 0.930 | 0.73 (0.51-1.05) | 0.091 | 0.92 (0.74-1.14) | 0.426 |
| Q3 | 1.03 (0.84-1.27) | 0.789 | 0.70 (0.47-1.04) | 0.077 | 0.92 (0.73-1.16) | 0.496 |
| Q4 | 0.92 (0.73-1.15) | 0.469 | 0.62 (0.40-0.97) | 0.038 | 0.72 (0.55-0.93) | 0.011 |
| *P* for trend |  | 0.495 |  | 0.027 |  | 0.019 |

Note: The "Non-depressed" trajectory group served as the reference outcome category. The model was adjusted for eGFR in addition to the covariates included in Model 3, namely age, sex, ethnicity, education level, marital status, smoking status, alcohol consumption, body mass index, moderate physical activity, hypertension, dyslipidemia, diabetes, arthritis, stroke, heart disease, and vitamin D.
